# LRP-1: Functions, Signaling and Implications in Kidney and Other Diseases

**DOI:** 10.3390/ijms151222887

**Published:** 2014-12-10

**Authors:** Ling Lin, Kebin Hu

**Affiliations:** Division of Nephrology, Department of Medicine, College of Medicine, Penn State University, 500 University Drive, Hershey, PA 17033, USA; E-Mail: llin1@hmc.psu.edu

**Keywords:** LRP-1, signaling, tPA, integrin, tyrosine phosphorylation, pathophysiology

## Abstract

Low-density lipoprotein (LDL)-related protein-1 (LRP-1) is a member of LDL receptor family that is implicated in lipoprotein metabolism and in the homeostasis of proteases and protease inhibitors. Expression of LRP-1 is ubiquitous. Up-regulation of LRP-1 has been reported in numerous human diseases. In addition to its function as a scavenger receptor for various ligands, LRP-1 has been shown to transduce multiple intracellular signal pathways including mitogen-activated protein kinase (MAPK), Akt, Rho, and the integrin signaling. LRP-1 signaling plays an important role in the regulation of diverse cellular process, such as cell proliferation, survival, motility, differentiation, and transdifferentiation, and thus participates in the pathogenesis of organ dysfunction and injury. In this review, we focus on the current understanding of LRP-1 signaling and its roles in the development and progression of kidney disease. The role and signaling of LRP-1 in the nervous and cardiovascular systems, as well as in carcinogenesis, are also briefly discussed.

## 1. Introduction

Low-density lipoprotein (LDL)-related protein-1 (LRP-1), or cluster of differentiation (CD) 91, is a type 1 transmembrane protein belongs to the LDL receptor family, which is implicated in lipoprotein metabolism and in the homeostasis of proteases and protease inhibitors [1,2,3,4]. It is also known as α2-macroglobulin receptor (α2MR) [4,5,6]. Huang, *et al.* [7] used matrix-assisted laser desorption/ionization-time-of-flight (MALDI-TOF) to analyze tryptic peptides of type V TGF-β receptor (TβR-V) purified from bovine liver, and found that LRP-1 is identical to TβR-V and mediates the growth inhibitory response to TGF-β1 and insulin-like growth factor-binding protein (IGFBP)-3. Thus, LRP-1 is also named as TβR-V [7]. Currently, LRP-1 has two known functions: (1) as a scavenger receptor to participate in the endocytosis of its numerous ligands; (2) as a signaling receptor to modulate various cellular processes [1,8,9]. The unique property of LRP-1 coupling endocytosis and signaling enable it to sense the ambient environment of the cells and tune the strength and breadth of the signaling and response [10].

Mature LRP-1 is derived from a 600-kDa precursor, which is subsequently cleaved by furin into a two-chain form consisting of an extracellular 515-kDa α subunit and an 85-kDa β subunit [4,11]. The extracellular α subunit consists of four ligand-binding domains (DI, DII, DIII, and DIV) and epidermal growth factor (EGF) repeats. LRP-1 interacts with more than 40 different ligands through its extracellular domain including tissue plasminogen activator (tPA) and connective tissue growth factor (CTGF) [8]. The 85-kDa β subunit consists of a transmembrane segment and cytoplasmic tail containing YxxL and dileucine motifs, two NPxY motifs, and numerous tyrosine residues [1,9,12]. The YxxL and dileucine motifs serve as principal endocytosis signals, whereas the NPxY motifs serve as secondary endocytosis signals and as binding sites for signaling adapter proteins [10]. Phosphorylation of the tyrosine residue(s) is essential for LRP-1 to relay its signal, though the exact mechanisms of the phosphorylation remain not complete understood. Our recent work demonstrated that phosphorylation of tyrosine (Tyr) 4507 is indispensable to LRP-1-mediated mitogenic signaling [13]. LRP-1 initiates signaling by direct ligand binding or transactivates signal pathways via its co-receptors [1,13,14,15,16,17,18,19,20,21,22,23,24,25,26,27].

Expression of LRP-1 is ubiquitous. Up-regulation of LRP-1 has been reported in numerous human diseases including Alzheimer disease [28,29], breast cancer [30], prostate cancer [31], multiple sclerosis [32], proliferative retinopathy [33], and ischemic cardiomyopathy [34]. Induction of LRP-1 and/or its ligands has also been observed in numerous animal models [14,20,35,36,37,38,39,40], suggesting that LRP-1 may act as a common receptor and its signaling plays an important role in the pathophysiology of human diseases.

## 2. Low-Density Lipoprotein (LDL)-Related Protein-1 (LRP-1) Signaling in Kidneys

**Figure 1:**
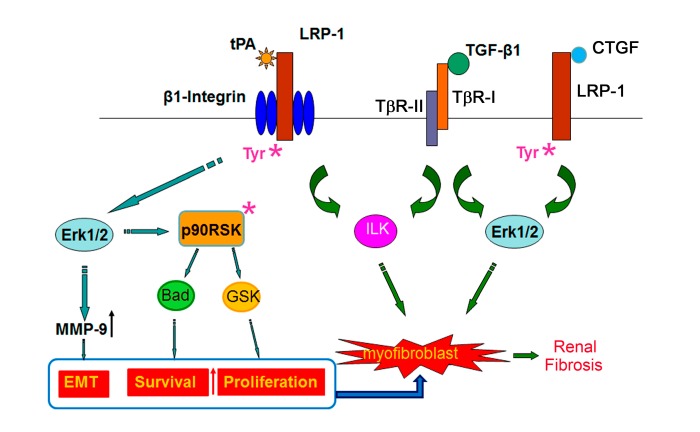
Fibroblast Low-density lipoprotein (LDL)-related protein-1 (LRP-1) signaling in renal fibrogenesis. Interaction of LRP-1 and its ligands mediates fibroblast differentiation and transdifferentiation. Tissue-type plasminogen activator (tPA) binds to LRP-1 and induces its tyrosine phosphorylation, followed by activation of: (1) extracellular signal-regulated kinases (Erk)1/2 pathway to stimulate matrix metalloproteinase (MMP)-9 production and trigger the epithelial mesenchymal transition (EMT); (2) p90 ribosomal S6 kinase (p90RSK) and Bad pathway to promote fibroblast survival; (3) p90RSK and glycogen synthase kinase (GSK)3β pathway to induce proliferation; (4) TGF-β1-mediated β1 integrin and integrin-linked kinase (ILK) pathway to induce myofibroblast activation. Connective tissue growth factor (CTGF) binds and induces LRP-1 tyrosine phosphorylation, and promotes TGF-β1-mediated Erk1/2 activation, which leads to synergistic activation of myofibroblasts. Figure was modified with permission [43]. * stands for phosphorylation.

In the obstruction-induced fibrotic kidneys, the expression of LRP-1, as well as many of its ligands including tPA [14,20] and CTGF [40], is markedly induced after obstructive injury, predominantly in the renal interstitial region, the site of most inflammatory infiltration and transdifferentiation of residential renal cells [14,20,40]. LRP-1 has been shown, at least *in vitro*, to mediate or modulate the profibrotic effects, or signal response, of several prominent profibrotic factors including tPA [13,14,19], TGF-β1 [41,42], and CTGF [24]. Thus, it is reasonable to speculate that LRP-1 serves as a common receptor of multiple profibrotic factors and mediates their profibrotic effects by activating various signaling cascades (Figure 1).

### 2.1. Tissue Plasminogen Activator (tPA)/LRP-1 Signaling

In general, tPA in the circulation is produced and maintained by vascular endothelial cells. However, our recent work in the chimerical mice, which were created by bone-marrow transplantation between wild-type and tPA knockout mice and lacked tPA in either the myeloid or parenchymal cells, demonstrated that myeloid cells are the major source of tPA induced in the fibrotic kidneys promoting fibrosis and inflammation, whereas plasma tPA has little effects [44]. Myeloid-derived tPA interacts with LRP-1 on various types of cells and activates multiple signaling cascades to modulate cellular differentiation and transdifferentiation to promote kidney fibrosis and inflammation.

Our previous work showed that tPA binds to LRP-1 on kidney fibroblasts and induces its β subunit Tyr 4507 phosphorylation and subsequent activation of extracellular signal-regulated kinases (Erk)1/2 mitogen-activated protein kinase (MAPK) [13,14]. Although the exact molecular detail remains unknown, tyrosine residues on the β subunit of LRP-1 provide docking sites for signaling adaptor protein including SHC-adaptor protein (Shc) [45,46,47], which upon phosphorylation will then recruit growth factor receptor-bound protein 2-son of sevenless (Grb2-Sos), and activate Ras signaling [47]. Because v-Src-induced Tyr4507 phosphorylation causes association of LRP-1 with the adaptor protein Shc [21,45], it is likely that Shc mediates Ras-Erk1/2 signal transduction of tPA and LRP-1. We have already demonstrated that tPA and LRP-1-induced Erk1/2 activation plays a pivotal role in fibroblast proliferation, survival, and transdifferentiation leading to the interstitial accumulation of myofibroblasts, fibroblasts and fibrosis [13,14,19,20] (Figure 1). Firstly, LRP-1-mediated Erk1/2 activation induces MMP-9 expression and production in kidney fibroblasts [14], which in turn degrades tubular basement membrane of epithelial cells and initiates the type 2 epithelial mesenchymal transition (EMT) [48,49], an important process that contributes to the size of activated fibroblast population and fibrogenesis [50,51,52,53,54]. Whether LRP-1 has a direct role in type 2 EMT remains unknown. However, LRP-1 has been shown to mediate Hsp90-induced type 3 EMT in prostate cancer cells [15]. Secondly, Erk1/2 activation directly phosphorylates and activates p90 ribosomal S6 kinase (p90RSK) [55,56,57], which, in turn, activates various signaling events through selection of different phosphorylation substrates including glycogen synthase kinase (GSK)3β and Bad [55,57]: (1) by phosphorylation and subsequent degradation of GSK3β, LRP-1 promotes fibroblasts into S phase of cell division and induces fibroblast proliferation and accumulation [13]; (2) by phosphorylation Bad, LRP-1 suppresses the cytosol release of cytochrome C from mitochondria, prevents the cleavage and activation of caspases, and promotes fibroblasts and myofibroblasts survival by blocking their apoptosis [19].

LRP-1 Tyr4507 phosphorylation also plays an essential role in its interaction with other signal pathways such as platelet-derived growth factor (PDGF) [21] and β1 integrin [20]. We have found that tPA-induced phosphorylation of LRP-1 β subunit triggers the recruitment of β1 integrin, which forms complex with LRP-1 leading to aggregation and clustering of β1 integrin. In the presence of TGF-β1, LRP-1-mediated β1 integrin signaling and its downstream integrin-linked kinase (ILK) are potentiated to full activation, resulting in myofibroblasts activation and excessive matrix production [20]. Thus, tPA interacts with fibroblast LRP-1 to promote fibrosis through multiple signal pathways to induce fibroblast activation, proliferation, and survival (Figure 1).

In addition to fibroblasts, tPA also interacts with macrophage LRP-1 to modulate their migration and accumulation in the injured kidneys [43,44,58,59]. Cao and colleagues have shown that tPA, together with PAI-1, forms complex with LRP-1 and integrin CD11b to promote macrophage migration [17]. We further elucidated that CD11b downstream focal adhesion kinase (FAK) is phosphorylated by tPA at Tyr925, which leads to the activation of Ras-related C3 botulinum toxin substrate 1 (Rac1) [44]. FAK may regulate cytoskeletal events through modulation of the paxillin kinase linker (PKL/G protein-coupled receptor kinase-interactor 2 (Git2)) and β–pix complex [60]. β–pix, as an exchange factor for cell division control protein 42 homolog (Cdc42), is connected to focal adhesions through binding of PKL/Git2 to paxillin [61], and also serves as a scaffold to activate Rac and p21-activated kinase (PAK) signaling [62]. FAK induces the tyrosine phosphorylation of β–pix, leading to the recruitment and activation of Rac1 and subsequent actin cytoskeleton rearrangement of and cell migration [63]. Intriguingly, LRP-1 also mediates tPA-induced M1 macrophage survival through a pathway involving p90RSK and p38 MAPK [58].

Of note, we have demonstrated that TGF-β1 stimulates mothers against decapentaplegic homolog 3 (Smad3) phosphorylation and activation in LRP-1 knockout fibroblasts [20]. However, *in vivo* data demonstrated that both smooth muscle [64] and macrophage-specific LRP-1-deficient [65] mice, in response to atherosclerotic injuries, display activated Smad2/3 signaling suggesting that LRP-1 down-regulates TGF-β1 signaling [66]. Thus, the *in vivo* role of LRP-1 in renal fibrosis is warranted to be further investigated.

### 2.2. Connective Tissue Growth Factor (CTGF)/LRP-1 Signaling

CTGF, a 36 to 38 kD cysteine-rich secreted protein, was identified as a ligand of LRP-1 in 2001 [67]. CTGF is generally considered as a downstream mediator of profibrotic factor TGFβ1, however, the study from Yang and colleagues [24] demonstrated that CTGF alone does not induce myofibroblast differentiation, but it markedly augments TGF-β1-mediated myofibroblast activation as indicated by *de novo* induction of smooth muscle actin alpha (αSMA) and extracellular accumulation of fibronectin. They further found that LRP-1 antagonist RAP inhibits CTGF-induced LRP-1 tyrosine phosphorylation and blockades its profibrotic effects, while TGF-β1-induced Smad2 phosphorylation and its association with Smad4 have little effect. Instead, CTGF activates Erk1/2 in kidney fibroblasts, and inhibition of Erk1/2 abolishes CTGF-mediated myofibroblast activation [24]. Thus, LRP-1-mediated Erk1/2 phosphorylation promotes fibroblast transdifferentiation into matrix-producing myofibroblasts (Figure 1).

## 3. LRP-1 Signaling in Nervous System

In response to injury, LRP-1 and its ligands such as tPA are also up-regulated in various cells of both central and peripheral nervous systems [10,38], suggesting an integral role of LRP-1 in the nervous system.

### 3.1. LRP-1 and Central Nervous System

Wang and colleagues have shown that LRP-1 mediates tPA-induced matrix metalloproteinase (MMP)-9 expression in human cerebral microvascular endothelial cells, and inhibitors of the transcription factors AP-1 and NF-κB suppress tPA effect [68]. Up-regulated MMP-9 subsequently promotes neuron death by matrix degradation and disruption of neuron integrity [69,70]. In a middle cerebral artery occlusion (MCAO)-induced brain ischemic model, Yepes and others have demonstrated that induction of endogenous tPA or injection of exogenous tPA induces a rapid and dose-dependent increase in vascular permeability resulting in opening of blood-brain barrier (BBB). They further showed that LRP-1 mediates BBB opening, since both LRP-1 antagonist receptor-related protein (RAP) and its neutralizing antibody block the activity [38]. Later, Yepes group also found that MCAO-induced microglial activation, as demonstrated ameboid morphology and double immune staining of β-isolectin and F4/80, as well as inflammatory markers such as inducible nitric oxide synthase (iNOS), in the wild-type mice, is significantly decreased in tPA−/− and microglia-specific LRP-1 knockout (macLRP−/−). In addition, administration of exogenous tPA induces microglial activation in tPA−/− mice but not in the macLRP−/− mice, suggesting that LRP-1 mediates tPA-induced microglial activation and inflammatory response in the ischemic brain. Although the exact signaling mechanism remains unknown, they have shown that LRP-1 promotes NF-κB signaling in this model [37].

Alzheimer disease (AD) is characterized by amyloid-β (Aβ) deposition in brain parenchyma as senile plaques and in cerebrovasculature as cerebral amyloid angiopathy [71]. Qiu Z. and colleagues found an 85% increase of LRP1 in human AD brain frontal cortex with concomitant increase of its ligands apolipoprotein E (apoE) and α2-macroglobulin (α2M) [28]. In another immunohistological study, it has been shown that neuron expression of LRP-1 is up-regulated and co-localizes with Aβ within senile plagues of AD patients [29]. LRP-1 has been shown to not only interact with Aβ precursor protein (APP) and regulate APP processing into Aβ [72,73] but also mediate Aβ export across the BBB [74,75,76]. Therefore, impaired LRP-1 function is implicated in the pathogenesis of AD. This is confirmed by the finding reported by Dr. Bu group, which demonstrated, in a conditional LRP-1-deleted mouse model, that LRP-1 is a major Aβ clearance receptor in cerebral vascular smooth muscle cells (vSMCs), and its malfunction contributes to Aβ accumulation and the pathogenesis of Alzheimer disease [71].

### 3.2. LRP-1 and Peripheral Nervous System

Emerging evidences showed that LRP-1 signaling also play a critical role in the regeneration of peripheral nervous system after injury. Schwann cells are the first responders to acute peripheral nerve injury [10]; their activation, proliferation and migration play an important role in establishing scaffolds for axonal regeneration [77]. In the injured peripheral nerve, LRP-1 is markedly induced in Schwann cells, and its signaling through Akt pathway promotes Schwann cell survival [78]. LRP-1 also has been shown to interact with different ligands and initiates unique signaling cascades to enhance Schwann cell migration: (1) LRP-1 interacts with MMP-9 via its hemopexin domain and promotes Schwann migration through a signal pathway involving Erk1/2 and Akt [79]; (2) LRP-1 interacts with tPA and α2M and initiates the activation of Rac1 to induce Schwann cells migration [22]. The pro-regenerative effect of LRP-1 signaling has also been confirmed in the Schwann cell-specific LRP-1 knockout mice, which showed exacerbated nerve injury accompanied by loss of motor and sensory function, resulting in the substantially attenuated regeneration after nerve injury [80].

In neurons, LRP-1 also acts as co-receptor of tropomyosin receptor kinase (Trk) receptor [23] or its NPxY motif interacts with adaptor protein postsynaptic density protein 95 (PSD95) to bridge the *N*-methyl-d-aspartate (NMDA) signaling [81,82] to mediate tPA-induced phosphorylation and activation of downstream Akt and Erk pathways, leading to neurite outgrowth.

## 4. LRP-1 Signaling in Cardiovascular Disease

In addition to its endocytosis function in lipoprotein metabolism and homeostasis of proteases involved in matrix modulation, LRP-1 also regulates the pathogenesis and progression of cardiovascular disease through various signaling mechanisms.

### 4.1. LRP-1 Signaling in Macrophages

Generally, macrophage LRP-1 is considered to protect against atherosclerosis, which has been verified in various models including macrophage LRP deficiency in either an LDL receptor knockout or apolipoprotein E/LDL receptor double knockout mice [65,83,84]. The possible underlying mechanisms include that macrophage LRP-1 promotes macrophage survival by activating Akt pathway and increases efferocytosis [85]; by binding to TGF-β2 to modulate TGF-β/Smad2/3 signaling, as well as PDGF receptor β [65], and reduces elastic lamina breaks by decrease MMP-9 expression [84].

### 4.2. LRP-1 Signaling in Muscle Cells

In consistent with the role of macrophage LRP-1 in atherosclerosis, study using mice with conditional deletion of LRP-1 in vSMCs also supports the protective effect of vSMC LRP-1, which showed that vSMC LRP-1-deficient mice display hyperplasia of aortal wall, disruption of elastic lamina, and formation of aortic aneurysm, and are highly susceptible to atherosclerosis [86]. The protective effect of vSMC LRP-1 is mediated through inhibition of PDGF receptor β phosphorylation [86], as well as a PDGF receptor β-independent mechanism involving the regulation of CTGF and a novel LRP-1 ligand high-temperature requirement factor A1 (HtrA1) [87].

However, *in vitro* studies indicate that muscle cell membrane LRP-1 appears to modulate some cellular processes involved in the pathogenesis of atherosclerosis and fibrosis such as inducing cell contraction and proliferation [16,88,89]. tPA has been shown to promote smooth muscle cell activation and increase the vessel tone [16,88]. tPA-mediated vasocontraction and calcium mobilization from intracellular stores require the formation of a complex between LRP and αvβ3 in vSMCs [16] suggesting a role of LRP-1-mediated integrin signaling. Stouffer and other found that activated α2M and TGF-β1 synergistically promote smooth muscle cell proliferation in LRP-1-dependent manner [89]. Although the details of the signaling remain unknown, studies from Branda group [41,42] demonstrated that decorin, a member of the small leucine-rich proteoglycan family, modulates TGF-β signaling by binding to LRP-1 and inhibiting TGF-β-dependent signaling and fibrotic response in skeletal muscle cells. Intriguingly, the modulatory decorin/LRP-1 pathway requires the activation of TGF-β-dependent Smad pathway and involves phosphatidylinositol-4,5-bisphosphate 3-kinase (PI3K) activity [42]. Additional, in a hind limb ischemia model, LRP-1 acts as the cytokine midkine (MK) receptor to support neutrophil adhesion and trafficking by promoting high affinity conformation of β2 integrin [90], suggesting a role of LRP-1 in muscle inflammation. Of note, *in vitro* mechanistic studies may not be transferrable into *in vivo* settings due to the complexity of cross-talks among various signal pathways and different cell types and organ systems.

### 4.3. LRP-1 Signaling in Fibroblasts

LRP-1 has been shown to modulate the production and remodeling of extracellular matrix components in fibroblasts [20,91], thus is implicated in the atherosclerogenesis. LRP-1 mediates cytosolic phospholipases A2 (cPLA2) phosphorylation and ATP-binding cassette, subfamily A, member 1 (ABCA1) expression to modulate cellular cholesterol export [92], and stimulates a canonical Wnt5a signaling pathway that prevents cholesterol accumulation, as well as promotes lipolysis and fatty acid synthesis through inhibition of GSK3β and its target acetyl-CoA carboxylase (ACC) [93]. We also have demonstrated that LRP-1 mediates tPA-induced p90RSK activation in fibroblasts [13,19], which has been shown to promote endothelial dysfunction and atherosclerosis in a diabetic model [94]. Thus, the exact *in vivo* role of fibroblast LRP-1 signaling in cardiovascular disease remains to be elucidated.

## 5. LRP-1 Signaling in Cancer

Although LRP-1 has been shown to be up-regulated in various cancers, its role and signaling in carcinogenesis and progression is context-dependent. Some studies indicated that LRP-1 facilitates tumor progression [18,95,96,97,98,99], while others showed that LRP-1 may have opposite effects [100,101]. LRP-1-mediated activation of FAK, Erk1/2 and Akt pathways can induce tumor cell proliferation, migration and invasion directly [18,97] or indirectly through MMP-2 and MMP-9 induction [95]. Intriguingly, Staudt and colleagues demonstrated that in a subcutaneous PanO2 pancreatic cancer isograft model, macrophage LRP-1 deficiency induces macrophage infiltration into tumor, expression of proinflammatory chemokines, and tumor angiogenesis [100].

## 6. Conclusions

It is clear that LRP-1 mediates various signaling pathways to modulate numerous cellular processes and play an important role in the pathogenesis and progression of human diseases. However, effects of LRP-1 signaling are context dependent and related to individual ligands and cell types. The dual functions of LRP-1 as receptor for endocytosis and signaling further complicate the interpretation of its actions and mechanisms. Challenges regarding LRP-1 in disease pathophysiology remain to be answered.
